# Evaluation of commercial veterinary probiotics containing enterococci for transferrable vancomycin resistance genes

**DOI:** 10.1186/s13104-020-05114-1

**Published:** 2020-06-04

**Authors:** Ana Berreta, Rachel M. Baumgardner, Jamie J. Kopper

**Affiliations:** 1 Department of Veterinary Clinical Sciences, College of Veterinary Medicine, Washington State University, Pullman, WA 99163 USA; 2 Present Address: Department of Veterinary Clinical Sciences, Iowa State University, Ames, IA 50010 USA

**Keywords:** Antimicrobial resistance, Enterococcus, Vancomycin, Probiotic

## Abstract

**Objective:**

Vancomycin resistant enterococci (VRE) are of significant public health concern. The identification of VRE in livestock and food has increased. The objective of this study was to determine if the transferrable vancomycin resistance genes *vanA* and *vanB* were present in probiotics marketed for use in animals that claimed to contain *Enterococcus* spp.

**Results:**

Of the 40 products selected, *Enterococcus* spp. DNA was successfully extracted from 36 products. Of these 36 products with enterococcal DNA, 2 (6%) had a PCR product consistent with *vanA* which was confirmed by sequencing. None of the products appeared to contain *vanB.*

## Introduction

The development of antimicrobial resistance (AMR) is considered one of the greatest threats to public health [1, 2]. Specifically, the development of vancomycin resistant enterococci (VRE) is of particular concern due to its association with significant morbidity and mortality in hospital acquired infections and few means to treat such infections [3]. Resistance in VRE is typically conferred by *vanA* or *vanB* genes, although other variants exist, both of which are transmissible by plasmids or transposons [4, 5].

Probiotics, defined as live micro-organisms that when administered in adequate amounts confer a health benefit to the host [6], are often considered “generally recognized as safe” within the United States of America (USA) meaning that they are exempt from regulatory testing by the Food and Drug Administration [7]. Thus, routine surveillance for AMR genes in commercial probiotics is not performed in the USA. *Enterococcus* spp. are frequently used in probiotics due to their reported potential to confer several health benefits to the host including restoration of normal microbiota following antimicrobial associated dysbiosis [8] and positive effects on the immune system [9]. However, the use of *Enterococcus* spp. in probiotics is controversial due to their association with nosocomial infections and potential to contain multiple AMR genes including vancomycin resistance genes [10, 11].

The objective of this study was to determine if the transferrable vancomycin resistance genes *vanA* and *vanB* were present in probiotics marketed for use in animals that claimed to contain *Enterococcus* spp. We hypothesized that commercially available veterinary probiotics containing enterococci would have the transferrable vancomycin resistance genes *vanA* and/or *vanB*.

## Main text

### Materials and methods

#### Selection of probiotics

A list of commercially available probiotics that claimed to contain at least one *Enterococcus* spp., were marketed for use in animals and available for purchase by owners was compiled using common online sources. A random number generator (http://www.random.org) was utilized to select 40 products for further evaluation. Products were purchased and stored according to the manufacturer’s recommendations.

#### DNA extraction from commercial probiotics

DNA was extracted from probiotics using one of several commercially available DNA extraction kits from QIAGEN (QIAGEN, Valencia, CA, USA) including the DNeasy PowerFood Microbial kit, DNeasy PowerSoil kit, DNeasy Blood and Tissue kit, QIAamp DNA Stool Mini kit and DNeasy PowerMax Soil kit. The kit utilized for each product is noted in Table 1. Due to the variability of probiotic substrates, multiple kits were utilized to find the best extraction method for each probiotic. DNA quantity and quality (i.e. A260:280 ratio) was assessed using NanoDrop^®^ spectrophotometry (Thermofisher, Waltham, MA, USA). Extractions with the highest quantity and best purity were used for further analyses.

**Table 1:** Extraction kits utilized for each product

Species	Product	Extraction kit	Ent PCR	*vanA*	*vanB*
Bovine	1	d	+	–	–
	2	c	+	–	–
	3	d	+	–	–
	4	e	+	–	–
	5	a	+	–	–
	6	a	+	–	–
	7	e	+	–	–
Camelid	8	b	–	–	–
Canine	9	b	+	–	–
	10	a	+	–	–
	11	b	+	–	–
	12	a	+	–	–
	13	d	+	–	–
	14	c	+	+	–
	15	a	+	–	–
	16	a	+	–	–
	17	a	+	–	–
Caprine	18	d	+	+	–
Equine	19	d	+	–	–
	20	d	+	–	–
	21	d	+	–	–
	22	c	–	–	–
	23	d	–	–	–
	24	d	+	–	–
	25	d	+	–	–
	26	d	+	–	–
	27	d	+	–	–
	28	e	+	–	–
	29	d	+	–	–
	30	e	+	–	–
	31	c	–	–	–
Multiple*	32	c	+	–	–
Feline	33	d	+	–	–
	34	a	+	–	–
	35	d	+	–	–
	36	a	+	–	–
	37	a	+	–	–
	38	b	+	–	–
	39	b	+	–	–
	40	a	+	–	–

Letters under the extraction kit column signify the QIAGEN ^®^ (Valencia, CA USA) extraction kit utilized: (a) DNeasy PowerFood Microbial Kit, (b) DNeasy PowerSoil Kit, (c) DNeasy Blood and Tissue Kit, (d) QIAamp DNA Stool Mini Kit, (e) DNeasy PowerMax Soil Kit. PCR’s marked as + were identified in that product PCR and for *vanA* and *vanB* the PCR product was confirmed to match the gene of interest by sequencing. Ent is indicative of *Enterococcus* spp.

*Product marketed for use in multiple animal species including horses, pigs, poultry and ruminants

#### Extraction of DNA from positive control bacteria for AMR genes

*Enterococcus avium* containing the *vanA* gene obtained from the Center for Disease Control and FDA Antibiotic Resistance Isolate Bank (Atlanta, GA, USA) and *Enterococcus faecalis* containing *vanB* (ATCC^®^700802) obtained from the American Tissue Type and Culture (Manassas, VA, USA) were used as positive controls. Frozen cultures were propagated according to the suppliers’ instructions. Subsequently, colonies were placed in 1.5 ml Tris–EDTA buffer and DNA was extracted using QIAGEN’s DNeasy Ultraclean Microbial kit (QIAGEN, Valencia, CA, USA) according to the manufacturer’s instructions. DNA concentration and quality were was assessed using NanoDrop^®^ spectrophotometry (Thermofisher, Waltham, MA, USA).

#### Bacterial DNA confirmation

The presence of bacterial DNA in each probiotic DNA extraction was confirmed using a bacterial 16S rRNA PCR as previously described (Table 2) [12] with the following modifications: amplification was performed by an initial denaturalization at 95 °C for 3 min, then 25 cycles of denaturalization at 95 °C for 30 s, annealing at 50 °C for 1 min, and extension at 72 °C for 2 min. The final extension was performed at 72 °C for 10 min, then the sample was held at 4 °C until ready to be analyzed via gel electrophoresis. The PCR products were evaluated by electrophoresis in 2% agarose gel, stained with ethidium bromide and visualized to evaluate for a corresponding 994 bp PCR product.

**Table 2:** Primers used for PCR analyses

Primers	Nucleotide sequence (5′-3′)	Size of PCR product (bp)
16S rRNA^12^	Forward: TGCCAGCAGCCGCGGTA (516f) Reverse: GGTTACCTTGTTACGACTT (1510r)	994
ent^13^	Forward: TACTGACAAACCATTCATGATG Reverse: AACTTCGTCACCAACGCGAAC	112
vanA^14^	Forward: AATACTGTTTGGGGGTTGCTC’ Reverse: CTTTTTCCGGCTCGACTTCCT	734
vanB^14^	Forward: GCGGGGAGGATGGTGGGATAGAG Reverse: GGAAGATACCGTGGCTCAAAC	420

#### Detection of *Enterococcus* spp. by PCR

Probiotic DNA was evaluated for the presence of DNA from *Enterococcus* spp. using previously published primers (Table 2) and PCR protocols [13]. PCR products were evaluated by electrophoresis in 2% agarose gel, stained with ethidium bromide and visualized for an appropriately sized PCR product. DNA samples with an appropriately sized PCR product were considered positive for *Enterococcus* spp. DNA.

#### Validation of *vanA* and *vanB* PCRs

Previously published PCRs for *vanA* and *vanB* [14] were used in this study (Table 2). The reactions were validated in our laboratory using DNA extracted from bacterial cultures with known *vanA* or *vanB* genes as described above. PCRs were confirmed to have DNA product of the expected size using gel electrophoresis. PCR products were confirmed to correspond with *vanA* and *vanB* sequences as described below.

#### Detection of *vanA* and *vanB* genes in probiotics via PCR

DNA from the probiotics with bacterial DNA were evaluated for the presence of *vanA* and *vanB* genes using previously published PCRs as described and validated above. The PCR products were evaluated by electrophoresis in 2% agarose gel, stained with ethidium bromide and visualized for an appropriately sized PCR product. For products without a corresponding band of interest, the PCR was repeated with the same parameters using 10 µl of the initial PCR as template. The second PCR was again evaluated by electrophoresis in 2% agarose gel, stained with ethidium bromide and visualized for an appropriately sized PCR product.

#### Sequencing of amplified AMR genes

Positive control and probiotic sample PCRs with appropriately sized PCR products were treated with ExoSAP-it reagent (Affymetrix Life Science Reagents, OH, USA) in accordance with the manufacturer’s instructions. Sequencing of the treated PCR products was performed using the corresponding forward primer and Big Dye 3.1 reagent mix (Applied Biosystems, Life Technologies, NY, USA). Sequence data were analyzed using Sequencher 5.2 software (GeneCodes Corp, MI, USA) and confirmed to represent the gene of interest by comparing the PCR product sequence to the known genetic sequence of the AMR gene of interest using the BLAST sequence analysis tool (https://blast.ncbi.nlm.nih.gov/Blast.cgi). Samples were considered positive for *vanA* or *vanB* if they had both PCR product size consistent with the gene of interest and the PCR sequence was confirmed to match that of the gene of interest.

### Results

*VanA* and *vanB* PCRs were successfully validated using the positive control isolates. Of the 40 products tested, all had evidence of bacterial DNA by 16S rRNA PCR. All the products claimed to contain at least one *Enterococcus* spp. on the label, however four of these products did not have evidence of *Enterococcus* spp. DNA based on this PCR, as shown in Table 1. The samples that did not have evidence of enterococcal DNA did not have *vanA* and *vanB* PCRs performed. Two probiotics had a PCR product consistent with *vanA* which was subsequently confirmed by sequencing. None of the products had PCR product consistent with *vanB*.

### Discussion

The AMR gene *vanA* was identified in 2 of the 36 products that contained *Enterococcus* spp. DNA in this study. None of the products contained *vanB*. Despite the relatively low incidence of detecting of *vanA* in these probiotics, given the public health significance of vancomycin resistance and the unregulated use of these products, we believe that these results are noteworthy.

*VanA* and *vanB* are the most prevalent vancomycin resistant variants in VRE [15, 16]. Consistent with the results from this study, *vanA* is more prevalent than *vanB* in food samples, which is likely due to increased mobility of the *vanA* gene cluster compared to *vanB* [17, 18]. Phenotypically, *vanA* is responsible for both a high level of resistance to vancomycin and teicoplanin [18]; both of which are considered medically important for use in resistant infections or in individuals allergic to β-lactams [4]. Furthermore, in addition to horizontal transfer to other *Enterococcus* spp., *vanA* has also been demonstrated to transfer to *Staphylococcus* spp. [19–22]. Thus, the identification of mobile elements conferring vancomycin resistance used in products that are marketed for use in companion animals (dogs) which live in close proximity with people and in minor food animal species (goats) is concerning.

Food products have been identified as a possible route for transfer of resistant *Enterococcus* spp. from animals to people [23–26] and the risk of transmitting VRE from animals to humans has been cited as a public health concern [27]. Probiotics are becoming increasingly popular in humans and animals stemming from a desire for more “natural” treatments, to decrease the use of antimicrobials and to improve gastrointestinal health [28, 29]. Given the nature that probiotics are administered—either in a daily fashion to prevent disease or in a daily fashion during intestinal disease—the presence of *vanA* is potentially concerning. In Europe, the food chain has been suspected to be a source of VRE acquired by humans [27] and the European Food Safety Authority has stated that commercial probiotics must establish their lack of acquired or transferrable resistance factors in order to be declared safe for human and animal consumption [30]. To date, similar requirements have not been implemented in the United States.

Interestingly, of the 40 products that claimed to contain *Enterococcus* spp. on the product label, only 36 had evidence of *Enterococcus* spp. DNA. These potential differences between the label claims and actual contents may supports previous studies that have repeatedly established discrepancies between label claims and viable micro-organisms in probiotics marketed for use in animals [29, 31]. Probiotics are typically considered “Generally Recognized as Safe” or GRAS in the United States by the Food and Drug Administration and typically do not undergo post-processing scrutiny or quality control. However, products containing *Enterococcus* spp. have been a source of debate in both the United States and Europe and do not automatically earn classification as GRAS in the US. However, several strains of *Enterococcus faecium* have been approved by the FDA for use in probiotics. The results from this study would suggest that further scrutiny is necessary. It is possible that strains that were once free AMR genes in starter cultures subsequently acquired such mobile elements and no longer represent the originally approved strain [32].

In conclusion, here we report identifying *vanA*, a known transferrable vancomycin resistant gene, in 2 of 36 commercially available probiotics marketed for use in animals which also contained *Enterococcus* spp. DNA. Given the public health significance of vancomycin resistance, the established transferable nature of *vanA* between *Enterococcus* spp. and other bacterial species and the growing popularity of probiotics we believe that these results warrant consideration.

## Limitations

There are several weaknesses of this study worth consideration. First, the identification of the *vanA* gene was determined using molecular based techniques, thus it is possible that despite results indicating the presence of *vanA* genes within these products, the bacteria would not be phenotypically consistent with vancomycin and teicoplanin resistant species. Such organisms have been reported rarely [33–35], however work by Gouisia and colleagues indicate that almost all *Enterococcus* spp. carrying *vanA* express corresponding resistance [17]. Second, the presence or absence of *Enterococcus* spp. was not confirmed by culture. Thus, it is possible that some of the products without *Enterococcus* spp. DNA had viable *Enterococcus* spp. that were not identified by the PCR due to low levels or other inhibiting factors. Likewise, it is possible that some products with *Enterococcus* spp. DNA identified by PCR did not have viable *Enterococcus* spp., and DNA from dead micro-organisms were amplified. Third, the results of this study are qualitative in nature (presence of absence of the gene) and not quantitative. Thus, although we identified the presence of *vanA* genes in veterinary probiotics, the quantity of genetic material per dose of probiotic was not measured and was beyond the scope of this work. Quantitative information may be valuable when determining the risk of conferring *vanA* resistance. Finally, given the lack of microbial culture data in this study, we were unable to determine which bacterial species contained the *vanA* gene. Further investigation is warranted to culture the bacteria in these products and determine MIC values for vancomycin to determine which bacteria have phenotypic resistance to vancomycin.

## Data Availability

The datasets used and/or analyzed during the current study are available from the corresponding author on reasonable request.

## Abbreviations

AMR: Antimicrobial resistance
PCR: Polymerase-chain reaction
USA: United States of America
VRE: Vancomycin resistant enterococcus
